# Prevalence of Stress and Perceived Contributing Factors Among Healthcare Workers at a District Health Center in Vietnam: A Mixed-Methods Study

**DOI:** 10.7759/cureus.115973

**Published:** 2026-09-08

**Authors:** Oanh Luu, Xuan Phan, Nguyet Ha, Thuy Hua

**Affiliations:** 1 Health Management Training Institute, Hanoi University of Public Health, Hanoi, VNM; 2 Outpatient Department, Long Dat Regional Health Center, Hanoi, VNM

**Keywords:** dass-21, depression, depression anxiety and stress scale (dass-21), healthcare workers, mixed-methods study, primary health care, psychological stress

## Abstract

Background: Psychological stress among healthcare workers (HCWs) is a growing concern in primary care settings, yet data from such facilities in Vietnam remain limited. This study aimed to estimate the prevalence of stress and explore perceived contributing factors among HCWs at a regional health center, a primary healthcare facility (formerly a district health center) in Vietnam.

Methods: A convergent mixed-methods study was conducted among all 167 HCWs at the Long Dat Medical Center in Ba Ria-Vung Tau Province, Vietnam, in 2025. The quantitative component used a self-administered questionnaire including the Depression, Anxiety and Stress Scale-21 Items (DASS‑21) stress subscale; stress severity was categorized as normal (0-14), mild (15-18), moderate (19-25), severe (26-33), and extremely severe (≥34). The qualitative component consisted of seven in-depth interviews and one focus group, analyzed thematically around individual, family-related, and health-facility-related factors.

Results: The mean DASS-21 stress score was 5.34 ± 6.31 (range 0-28). Overall, 74.8% (n=125) of HCWs had normal stress levels, while 11.4% (n=19) had mild, 11.4% (n=19) moderate, and 2.4% (n=4) severe or extremely severe stress, indicating that 25.2% (n=42), approximately one in four, experienced elevated stress symptoms. The most frequently reported manifestations were difficulty relaxing and feeling a high level of nervous energy. Qualitative findings showed that stress was driven by the interaction of financial strain, caregiving responsibilities, workload, and role overload, inadequate infrastructure and supplies, perceived imbalance between effort and remuneration, and tensions in workplace relationships, with family and collegial support acting as partial buffers.

Conclusions: About one in four HCWs at this primary healthcare facility experienced elevated stress, highlighting stress as an important workforce health issue rather than a marginal problem. Interventions should combine routine stress screening and targeted support for at-risk staff with organizational measures to improve working conditions, clarify roles, optimize workload and staffing, and strengthen fair and transparent incentive and support mechanisms.

## Introduction

Psychological stress among healthcare workers (HCWs) is a major global concern due to its impact on well‑being, quality of life, and the capacity to provide safe care. HCWs are frequently exposed to high workloads, emotionally demanding clinical situations, and complex interpersonal interactions, which can lead to elevated levels of general stress that extend beyond the workplace. During and after the COVID‑19 pandemic, studies using the Depression, Anxiety and Stress Scale‑21 Items (DASS‑21) have reported substantial proportions of HCWs with at least mild-to-moderate stress symptoms, underscoring a significant burden of psychological distress in this group [1-4].

In Vietnam, HCWs face additional pressures related to high patient volumes, limited resources, and increasing administrative demands. Local research using the DASS‑21 has documented notable prevalence of stress symptoms among health workers, including hospital staff and primary HCWs, with reported stress rates commonly around 15%-30% and significant associations with workload, income, workplace infrastructure, and perceived support. However, most existing evidence comes from hospital or provincial settings, while less is known about stress among HCWs working in primary care facilities [5-9].

Primary healthcare facilities form the backbone of the healthcare system in Vietnam, providing a large share of routine outpatient and inpatient services. HCWs at this level often work with constrained infrastructure, equipment shortages, and multiple overlapping responsibilities, which may contribute to sustained psychological stress. The Long Dat Health Center in Ba Ria-Vung Tau Province, Vietnam, is a primary healthcare facility providing both curative and preventive services. To address the evidence gap, this mixed‑methods study aimed to estimate the prevalence of stress among HCWs at the Long Dat Regional Medical Center using the stress subscale of the DASS‑21 and to explore their perceived contributing factors [7,9-11].

## Materials and methods

Study design and setting

A mixed-methods cross-sectional study was conducted at the Long Dat Regional Medical Center, a primary healthcare facility providing both curative and preventive services in Ba Ria-Vung Tau Province, Vietnam. The study was conducted from August 2024 to November 2025. Quantitative and qualitative data were collected between May and June 2025.

Sample size and sampling

For the quantitative component, a census approach was used: all 167 HCWs employed at the Long Dat Medical Center during the study period who met the inclusion criteria and consented were invited to participate in the survey.

HCWs who had worked at the Long Dat Regional Medical Center for at least three months at the time of data collection were eligible for inclusion. Those who were absent during data collection due to maternity leave, unpaid leave, or training programs of six months or longer, as well as research team members involved in data collection, were excluded.

For the qualitative component, purposive sampling was applied to select HCWs with variation in profession, department, gender, age, and seniority; seven in‑depth interviews and one focus group were conducted, consistent with common guidance that small, information‑rich samples are adequate for phenomenological inquiry and thematic saturation in relatively homogeneous populations.

Study variables and measurement

For the quantitative component, the study variables were grouped into two domains: individual characteristics and stress status. Individual variables included age, sex, marital status, educational level, income, and chronic disease status.

Stress was assessed using the Vietnamese version of the DASS-21, a widely used instrument that has been applied in previous studies in Vietnam and demonstrated acceptable psychometric properties. Stress was assessed using the stress subscale of the DASS-21, which comprises seven items (items 1, 6, 8, 11, 12, 14, and 18) [12,13]. Each item was scored from 0 to 3 according to symptom severity and frequency, and the summed score for the seven stress items was multiplied by two in accordance with DASS-21 scoring guidelines. Stress severity was categorized as normal (0-14), mild (15-18), moderate (19-25), severe (26-33), and extremely severe (≥34).

Qualitative themes

For the qualitative component, the interview guide was developed around three broad themes of perceived factors contributing to stress: individual factors, family-related factors, and health-facility-related factors. These themes were used to explore participants’ experiences and perceptions regarding the sources and context of stress in their daily work and personal lives.

Data analysis

Quantitative data were managed and analyzed using STATA, version 15.0 (StataCorp LLC, College Station, TX, USA). Qualitative data were audio-recorded, transcribed, coded into themes, and analyzed thematically.

Ethics in research

An institutional ethics committee approved the study for biomedical research under an official decision issued in May 2025. The Institutional Review Board of Hanoi University of Public Health issued approval (approval number: 160/2025/YTCC-HD3). Institutional permission was obtained from the participating hospital before data collection. All participants provided written informed consent.

## Results

Characteristics of the study participants

Table 1 presents the general characteristics of the study participants (n = 167). The majority of participants were aged 31-40 years (n=73, 43.7%), followed by those aged ≥41 years (n=64, 38.3%), with a mean age of 38.6 years (SD: 8.2; range: 25-59 years). Females accounted for a large proportion of the sample (n=124, 74.2%).

**Table 1:** General characteristics of study participants (n = 167) VND: Vietnamese dong

No.	Variable		Frequency	Percentage %
1	Age group	≤ 30 years	30	18
		31-40 years	73	43.7
		≥ 41 years	64	38.3
2	Sex	Male	43	25.8
		Female	124	74.2
3	Marital status	Single	23	13.8
		Married	135	80.8
		Divorced/Separated/Widowed	9	5.4
4	Education level	Secondary or lower	23	13.8
		College/Associate degree	43	25.8
		Bachelor’s degree	86	51.4
		Postgraduate	15	9
5	Years of work experience	≤ 10 years	61	36.5
		11-15 years	39	23.4
		>15 years	67	40.1
6	Personal income	< 10 million VND/month	122	73.1
		≥ 10 million VND/month	45	26.9
7	Chronic disease	Yes	24	14.4
		No	143	86.6

Most participants were married (n=135, 80.8%), while 13.8% were single and 5.4% (n=9) were divorced, separated, or widowed. Regarding education, more than half of the participants held a bachelor’s degree (n=86, 51.4%), followed by college/associate degree (n=43, 25.8%), while a smaller proportion had postgraduate qualifications (n=15, 9.0%) or secondary education or lower (13.8%).

In terms of work experience, 40.1% (n=67) had more than 15 years of experience, and 36.5% (n=61) had ≤10 years, with a mean duration of 13.5 years (SD: 8.7; range: 1-39). The majority of participants reported a personal income of less than 10 million Vietnamese dong (VND) per month (n=122, 73.1%).

Additionally, 14.4% (n=24) of participants reported having a chronic disease, while the majority (n= 143, 86.6%) did not.

Stress levels among HCWs at the Long Dat Health Center

Table 2 presents the distribution of stress symptoms among HCWs based on the DASS-21 items [12,13]. The results show that most HCWs reported low levels of stress symptoms across all items. The majority of participants selected “not at all” or “somewhat” for all statements, indicating that severe stress symptoms were relatively uncommon.

**Table 2:** Stress symptoms among healthcare workers (n=167)

Item	Not at all		Somewhat		Quite a lot		Very much		Mean	SD
	n	%	n	%	n	%	n	%		
I found it hard to relax.	88	52.7	62	37.1	13	7.8	4	2.4	0.60	0.74
I tended to overreact to situations.	133	79.6	31	18.6	3	1.8	0	0.0	0.22	0.46
I felt that I was using a lot of nervous energy.	91	54.5	55	32.9	16	9.6	5	3.0	0.61	0.78
I found myself getting agitated.	125	74.9	37	22.2	5	3.0	0	0.0	0.28	0.51
I found it difficult to relax.	117	70.1	42	25.1	8	4.8	0	0.0	0.35	0.57
I was intolerant of anything that kept me from getting on with what I was doing.	117	70.1	39	23.4	8	4.8	3	1.8	0.38	0.67
I felt that I was rather touchy.	135	80.8	26	15.6	6	3.6	0	0.0	0.23	0.50

Among the items, “I felt that I was using a lot of nervous energy” (mean = 0.61 ± 0.78) and “I found it hard to relax” (mean = 0.60 ± 0.74) had the highest mean scores, suggesting these were the most commonly reported stress-related experiences. In contrast, “I tended to overreact to situations” had the lowest mean score (0.22 ± 0.46), indicating that excessive emotional reactions were less frequently reported.

The overall mean stress score was relatively low (5.34 ± 6.31), with a wide range (0-28), reflecting variability in stress levels among participants.

Figure 1 illustrates the distribution of stress levels among HCWs. The majority of participants were classified as having normal stress levels (n=125, 74.8%). Mild and moderate stress levels were observed in equal proportions (n=19, 11.4% each), while only a small percentage of participants experienced severe or extremely severe stress (n= 4, 2.4%).

**Figure 1:**
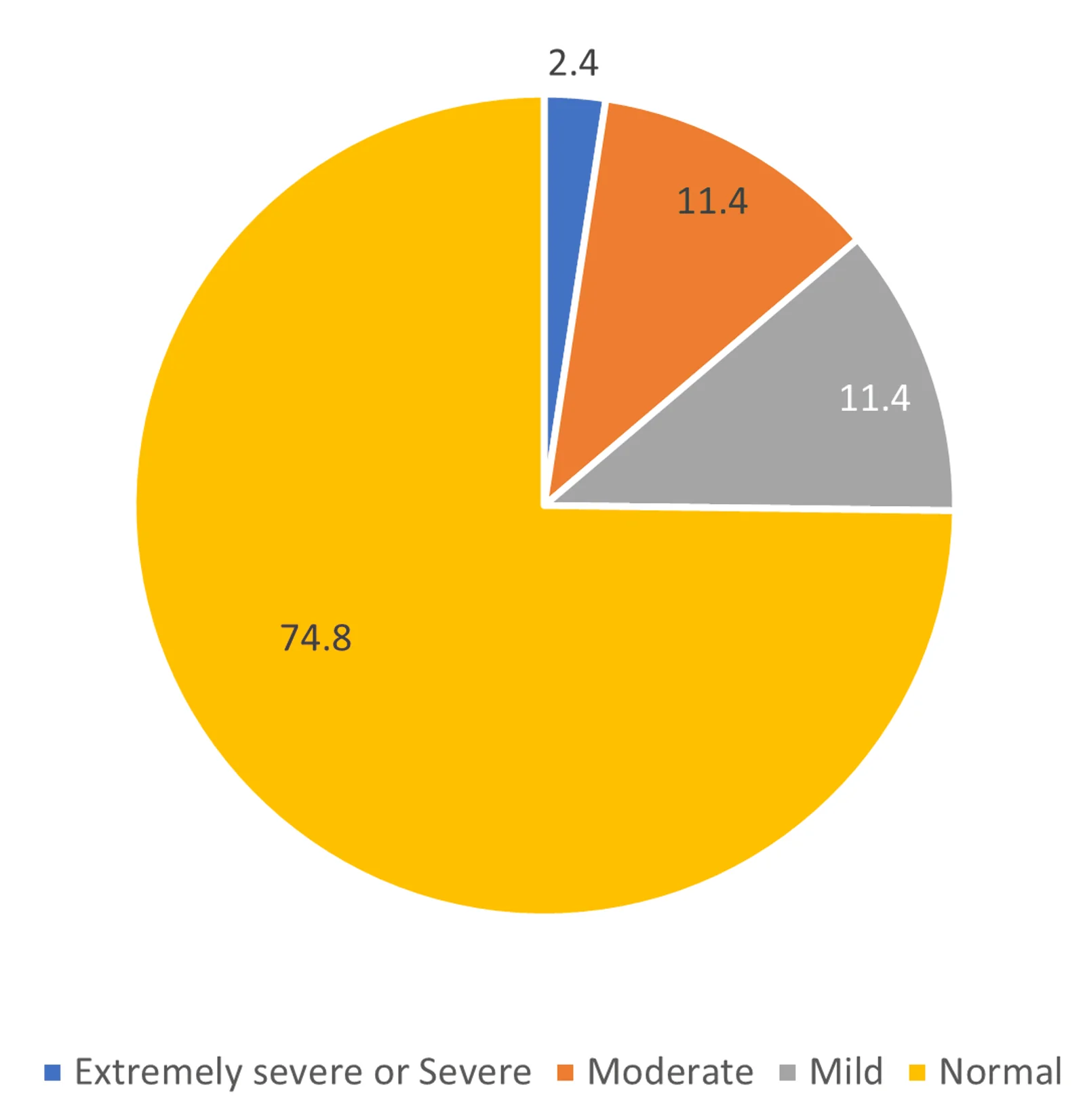
Percentage distribution of stress levels among healthcare workers (n=167)

Overall, although most HCWs were within the normal range, approximately one-quarter of participants exhibited some level of stress, indicating that stress remains a notable concern in this population.

Perceived factors contributing to stress among HCWs

Qualitative findings identified several perceived contributing factors to stress among HCWs, encompassing individual, family-related, and health system factors that often interacted in complex ways.

Individual-Related Factors

At the individual level, financial pressure emerged as a prominent source of stress. Many participants reported that income at the district health center was relatively low compared to higher-level facilities and insufficient to meet rising living costs. One participant stated, “income is the main issue for us… it is lower than staff at higher-level facilities, and inflation has increased in recent years”. This financial strain was perceived as particularly burdensome for female staff, who often had additional responsibilities related to childcare.

Work experience and professional expectations also contributed to stress. Participants noted that more experienced staff were often assigned greater responsibilities, leading to increased pressure. One respondent explained that “those who have worked longer are often given more responsibilities and therefore experience greater pressure”. Similarly, HCWs with higher educational qualifications, particularly physicians and those with postgraduate training, expressed dissatisfaction when their income and working conditions did not match their expectations. As highlighted by one participant, “those with higher qualifications expect better income and benefits, but this is difficult to achieve in a district-level facility”.

In addition, the availability of social support was identified as an important perceived contributing factor to stress among HCWSs. Participants who lacked emotional support at home reported higher stress levels, whereas those who could share their concerns with family members felt better able to cope. One participant noted that “having someone to talk to at home helps reduce stress”.

Family-Related Factors

Family responsibilities were identified as both a source of stress and a potential buffer, depending on the context. Participants with young children or multiple caregiving responsibilities reported greater stress due to competing demands between work and family life. As one participant described, “those with more children or young children are more easily distracted and therefore more stressed… especially when also caring for elderly or sick family members”.

Financial responsibilities within the family also played a significant role. Being the primary income earner was associated with increased psychological pressure, particularly among male participants who felt responsible for supporting their families. One participant shared, “I feel financial pressure as the main breadwinner while also taking care of my elderly parents”.

At the same time, family support could mitigate stress. Living with extended family members, such as grandparents, was described as beneficial in terms of childcare and household responsibilities. For example, one participant noted that “living with grandparents helps with childcare and reduces daily burdens”. These findings highlight the dual role of family factors, acting both as stressors and as sources of support.

Health System-Related Factors

At the organizational level, multiple factors related to the health system were reported to contribute to stress. Working conditions, including inadequate infrastructure, limited medical equipment, and shortages of supplies, were frequently mentioned. One participant expressed frustration, stating that “equipment and supplies are sometimes lacking, and it takes time to obtain them”. Poor physical working environments, such as cramped spaces and insufficient cooling systems, were also reported to negatively affect comfort and increase fatigue.

Compensation and financial mechanisms within the health center were another major concern. Participants perceived that remuneration was not commensurate with workload and contributions, particularly in revenue-generating departments. As one respondent noted, “the income distribution is rather equal instead of reflecting actual workload and effort”. Furthermore, income instability following the COVID-19 pandemic and reduced funding for preventive programs were identified as additional stressors.

Workload and job demands were also important contributors to stress. HCWs described increased pressure during disease outbreaks, frequent on-call duties, and the need to balance both clinical and preventive roles in a multi-functional health center. As one participant explained, “during outbreaks or periods of high workload, stress levels increase significantly”. Managers, in particular, reported high levels of stress due to the dual burden of administrative and professional responsibilities, as well as strict financial regulations.

Interpersonal relationships within the workplace also emerged as an important perceived contributing factor to stress among HCWs. While some participants highlighted positive aspects such as transparent evaluation processes and clear job assignments, others reported tension in relationships with colleagues or supervisors. As noted in a group discussion, “there are still cases of strained relationships with colleagues or leaders, which can create stress at work”.

## Discussion

Stress levels among HCWs

In this study, 25.2% (n=42) of HCWs at the Long Dat Medical Center had elevated stress levels on the DASS-21, meaning that approximately one in four staff members experienced at least mild stress symptoms. Although 74.8% (n=125) of participants were classified in the normal range, the finding that one quarter of the workforce already showed signs of stress is clinically and operationally important in a primary care facility that depends on a relatively limited human resource pool. The overall mean stress score was 5.34 ± 6.31, with scores ranging from 0 to 28, indicating that while severe symptoms were uncommon, stress was unevenly distributed and meaningfully present in a substantial subgroup of workers [14].

This proportion is consistent with the broader literature showing that DASS-21-measured stress among HCWs commonly affects around one-fifth to nearly one-half of staff, depending on the setting and period of assessment. In Vietnam, one study among HCWs reported stress levels of 10.7% mild, 8.7% moderate, 5.6% severe, and 2.9% extremely severe, which together also indicate that about one quarter of staff experienced stress symptoms. Other Vietnamese and regional studies conducted during high-pressure service periods likewise found substantial levels of stress among HCWs, particularly in hospital-based and frontline settings. Taken together, these comparisons suggest that the prevalence observed in the present study should not be regarded as trivial; rather, it indicates a meaningful burden of stress among HCWs in a primary healthcare context [6,14-16].

The item-level findings further clarify the nature of stress symptoms in this population. The highest mean scores were observed for symptoms related to nervous tension and difficulty relaxing, whereas over-reactivity was less commonly reported. This pattern is compatible with the conceptualization of the DASS-21 stress subscale, which primarily captures persistent tension, irritability, and difficulty unwinding rather than acute emotional outbursts. Even when these symptoms remain in the mild or moderate range, they may still affect concentration, sleep, energy, and emotional regulation in everyday work. Accordingly, the present findings suggest that stress among these HCWs may often manifest as ongoing internal strain rather than overt behavioral disruption [15,17-19].

From a service-delivery perspective, the finding that one in four HCWs had stress symptoms deserves careful attention. Elevated DASS-21 stress scores have been associated with poorer quality of life, reduced psychological well-being, and higher vulnerability to other mental health problems in healthcare personnel. In small or medium-sized primary care organizations, even a moderate burden of stress may have consequences for staff morale, teamwork, continuity of care, and long-term workforce retention. These results therefore support the need for regular screening, early supportive interventions, and organizational strategies that can prevent mild or moderate stress from progressing into more severe and persistent distress [3,9,14-16,20].

Perceived factors contributing to stress among HCWs

The qualitative findings provide additional context for understanding stress experiences among HCWs in this study. While most participants had stress scores within the normal range, approximately one in four reported elevated DASS-21 stress symptoms. The qualitative data identified several perceived contributing factors, including personal, family-related, and organizational challenges. These findings are broadly consistent with previous research suggesting that healthcare worker stress is shaped by multiple interacting factors [3,14,16].

At the individual level, financial pressure and unmet professional expectations were frequently described by participants as sources of stress. Participants reported that income at the Long Dat Regional Medical Center was often perceived as insufficient relative to living costs and lower than that available at higher-level facilities, particularly among staff with greater responsibilities or advanced qualifications. Similar concerns have been reported in studies conducted among HCWs in both Vietnam and other countries, although many of these studies were undertaken in hospital settings rather than primary healthcare facilities [6,14,16]. Participants also highlighted the importance of family support in coping with work-related stress, which is consistent with previous evidence suggesting that social support may play a protective role in healthcare settings.

Organizational and health system factors were also commonly mentioned during interviews. Participants described challenges related to infrastructure, equipment, and supply shortages, physical working conditions, workload, on-call duties, and the need to balance clinical, preventive, and administrative responsibilities. These perceptions are similar to findings reported in previous studies of HCWs, although differences in organizational context between primary healthcare facilities and hospitals should be considered when making comparisons [13,21,22]. Managers additionally described pressures associated with financial regulations and administrative responsibilities.

Participants also discussed concerns related to income distribution and workplace relationships. Some felt that compensation did not adequately reflect workload or effort, while others described tensions with colleagues or supervisors as contributing to stress experiences. These perceptions are consistent with literature suggesting that organizational justice, reward systems, and interpersonal relationships may influence psychological well-being among HCWs [6,14,16]. At the same time, participants noted positive aspects of the work environment, including clear task allocation and transparent performance evaluation. Together, these findings illustrate the range of factors that participants perceived as contributing to stress in this setting.

Taken together, the qualitative findings provide additional insight into the range of factors that participants perceived as contributing to stress, including individual, family-related, and organizational challenges. Although the study design does not permit conclusions regarding causality, the finding that approximately one in four HCWs reported elevated stress symptoms suggests that psychological well-being may be an important consideration in this setting. These findings may help inform future workplace health initiatives and support the development of strategies aimed at promoting staff well-being in primary healthcare facilities [3,9,14].

Limitations

This study has several limitations. First, the cross-sectional design precludes establishing causal relationships between potential contributing factors and stress. Second, stress was measured using a self-reported DASS-21 subscale rather than a clinical diagnosis, and responses may be affected by recall or social desirability bias. Third, data were collected from a single regional medical center with 167 HCWs, which may limit generalizability to other primary care settings with different contexts

## Conclusions

Stress remains an important occupational health concern among healthcare workers in primary healthcare settings. Interventions addressing organizational and personal stressors may help improve staff well-being and workforce sustainability. The most frequently reported symptoms were difficulty relaxing and experiencing high nervous energy, while severe stress was less common. Qualitative findings identified several perceived contributing factors to stress, including financial strain, caregiving responsibilities, workload and role overload, inadequate infrastructure and supplies, and perceived imbalance between effort and remuneration. Participants also highlighted the potential importance of family and workplace support in shaping their stress experiences. These findings suggest that routine attention to HCWs’ psychological well-being and workplace conditions may be beneficial in primary healthcare settings. Further studies in multiple settings are needed to better understand factors associated with stress and to inform appropriate interventions.
